# Physicochemical characteristics of sheep and goat pâtés. differences between fat sources and proportions

**DOI:** 10.1016/j.heliyon.2019.e02119

**Published:** 2019-07-27

**Authors:** Alfredo Teixeira, Samanta Almeida, Etelvina Pereira, Fernando Mangachaia, Sandra Rodrigues

**Affiliations:** Escola Superior Agrária/Instituto Politécnico de Bragança, Campus de Santa, Mountain Research Centre (CIMO), Apolónia., 5300-253, Bragança, Portugal

**Keywords:** Food science, Food quality, Food processing, Food analysis, Chemical food analysis, Food composition, Chemical composition of food, Food chemistry, Pâté, Goats and sheep meat, Pork fat, Olive oil, Fatty acid profile

## Abstract

The physicochemical composition of sheep and goat pâtés with different sources and percentage of fat (10% and 30%; pork belly or olive oil) were evaluated. A low-fat content (9.7–18.2%) was observed in the pâtés comparing with similar meat products. Cholesterol was lower in pâtés with olive oil than with pork fat. The source of fat (pork belly or olive oil) and the proportion of fat influenced significantly the fatty acid profile. Pâtés with olive oil have lower saturated fat content and highest monounsaturated fat while and goat meat pâtés have higher polyunsaturated fat content The polyunsaturated versus saturated fatty acids ratio varying from 0.21 to 0.39 and the total unsaturated fatty acids showed that sheep and goat pâtés are balanced products and could be an interesting way to the added value of animals with low commercial and consumer acceptability.

## 1 Introduction

The value of goat and sheep meat is affected by the seasonal availability of live animals, particularly those having quality brands, as Protected Designation of Origin (PDO) or Protected Geographical Indication (PGI). The highest prices of carcasses coincide with religious festivities as Christmas and Easter or other ethnic holiday dates as well as during summer parties. However, some animals, particularly those ones that come out of quality brands, with high carcass weight, have very low consumer acceptability and consequently have low commercial value. According to Webb et al. (2005) commercial value may be increased through production practices or meat processing through drying, curing with salts or smoking. This type of meat processing has been carried out by several researchers, in many countries, mainly from the Mediterranean area and the Middle East, Africa or South America. In Spain “Cecina” (Molinero et al., 2008), in Italy “violino di capra” (Fratianni et al., 2008) or in Brazil “Buchada” (Madruga et al., 2007) are examples of processed products formulated using goat or sheep meat as well as viscera (liver, heart, lungs, kidney), intestines and blood. Also, Dalmás et al. (2011) studied a goat pâté, prepared using variety meat (spent goat meat, goat liver and blood) and Guerra et al. (2011) evaluated a goat mortadella prepared with different levels of fat and goat meat from discarded animals.

Even though all mixture of cooked ground meat and fat minced into a spreadable paste is popularly known as pâté, as a meat processed product, Pâté is made from fresh livers and the most famous is the *Pâté de fois gras* produced with livers of fatty geese or duck. Other popular pâtés are commonly made with chicken and pork livers. But the use of other kinds of meat as sheep and goat is not so common. So, there is potential to differentiate individual market niches for processed meat from sheep and goats, but descriptions of the properties of the resulted products, which may be unique to consumers, will be required. Previously studies using the olive oil as fat replacement in meat products were made in venison sausages (Utrilla et al., 2014) in pork pâté (Domínguez et al., 2016) or in frankfurter sausages (Domínguez et al., 2017). Using sheep or goat meat comparing the pork fat or olive oil as fat sources a sensory analysis results on these pâtés was already published (Rodrigues et al., 2019). As far as we know the use of olive oil as fat replacement source in sheep or goat meat pâtés has not been studied so far.

In the present study, samples of eight types of pâtés were evaluated. The influence of species, fat source and fat percentage on (i) physical and (ii) chemical characteristics was analysed. We used mixed models' analysis to track differences (if any) between pâtés made with sheep and goats’ meat, and with 10% and 30% percentage olive oil or pork belly. Also, interactions were tested.

With H_0_ as substantial equivalence between the types of pâtés, the following hypotheses were tested:

H1 The pâtés have different physicochemical characteristics, particularly fatty acid profile.

(a).Sheep and goats’ pâtés have different physicochemical characteristics, particularly fatty acid profile.

(b).Olive oil and pork belly pâtés have different physicochemical characteristics, particularly fatty acid profile.

(c).Ten and 30% fat pâtés have different physicochemical characteristics, particularly fatty acid profile.

(d).Significant interaction between previous factors exist.

## 2 Material and methods

This work is part of a project between a research centre (Carcass and Meat Quality and technology Laboratory of Agriculture Scholl of Polytechnic Institute of Bragança), two Portuguese breeder associations (ANCRAS – National Breed Producers Association of Serrana Goat and ACOB – National Breed Producers Association of Bragançana Sheep) and a Bísaro breed producer and meat manufacturing industry (Bìsaro Salsicharia Tradicional).

The global objective was to develop new meat products to give added value to animals and carcass meat trimmings with low commercial value. Previous results on cured legs of goat and sheep, one of the new products, have already been published (Teixeira et al., 2017).

### 2.1 Pâté manufacturing process and sampling

Meat trimmings cleaned from subcutaneous and intermuscular fat, including only muscle tissue of carcasses used to manufacture the pâtés were from local breeds Churra Galega Bragançana ewes and Serrana goats with ages ranged between 5 and 7 years old, and with an average carcass weight of 20 kg. The pork belly is from females of local breed Bísaro which weighted between 110 and 130 kg body weight. Two lots of eight types of pâtés were manufactured in different places, one at the Laboratory of Carcass and Meat Quality of Agriculture School of Polytechnic Institute of Bragança and the other at a meat manufacturing industry Bísaro Salsicharia. Pâtés were made mincing and mixing meat (sheep or goat), previously boiled (60 min) with different amounts of pork belly/olive oil and other ingredients (water, milk, and mix) depending on the batch (see Table 1). Glass beakers of three hundred ml of capacity were filled with the mixture and sealed with a vacuum cap and heated for 30 min in a water bath in an oven at 170 °C. Beakers were refrigerated at 4 °C until analysed.

**Table 1:** Pâtés formulations.

Ingredients	GOO1	GOO3	GPB1	GPB3	SOO1	SOO3	SPB1	SPB3
Goat meat (g)	2180	1580	4500	3500	0	0	0	0
Sheep meat (g)	0	0	0	0	2180	1580	4500	3500
Pork belly (g)	0	0	500	1500	0	0	500	1500
Mix-088 Patê Bueton (g)	164	164	375	375	164	164	375	375
Cooking milk (ml)	200	200	500	500	200	200	500	500
Water (ml)	100	100	500	500	100	100	500	500
Olive oil (ml)	300	900	0	0	300	900	0	0
Total	2944	2944	6375	6375	2944	2944	6375	6375

Two replications of the eight types of pâtés: **GPB1** (Goat +10% pork belly); **GPB3** (Goat +30% pork belly); **GOO1** (Goat +10% olive oil); **GOO3** (Goat +30% olive oil); **SPB1** (Sheep +10% pork belly); **SPB3** (Sheep +30% pork belly); **SOO1** (Sheep +10% olive oil) and **SOO3** (Sheep +30% olive oil) were manufactured. For each lot of each replication, 3 samples were randomly selected from each type of pâté and each sample was analysed in triplicate, a total of 48 samples was analysed.

Olive oil presented the following fatty acids profile: 11.2% C16:0, 0.2% C17:1, 3.3% C18:0, 75.2% C18:1n-9, 7.7% C18:2n-6, 0.4% C20:0, 0.8% C18:3n-3, 0.2% C20:1n-9, 0.1% C22:0 according to the fatty acid profile analysis performed by Teixeira (2015) to the commercial brand used.

Pork belly fat presented the following fatty acids profile: 1.3% C14:0, 22.3% C16:0, 2.1% C16:1, 11.9% C18:0, 41.9% C18:1n-9, 15.7% C18:2n-6, 1.2% C18:3n-3, 0.78% C20:1n-9 (Teixeira, 2018).

### 2.2 Physicochemical analysis

The measurement of pH was performed according to the Portuguese standard NP 3441 (2008). Water activity was assessed according to AOAC (1990). Moisture, ashes, protein and hydroxyproline determination of collagen content and concentration were quantified according to the Portuguese standards NP 1614 (2009), NP 1615 (2002), NP 1612 (2002), and NP 1987 (2002), respectively. The cholesterol determination was carried out the procedure described by Domínguez et al. (2015b). Analysis of samples was carried out using a HPLC Ultimate 3000, Thermo Scientific system from Dionex (Germany).

### 2.3 Total fat and fatty acid profile

Total lipids were extracted from 25 g of ground meat sample, according to Folch et al. (1957) procedure. Fifty milligrams of fat were used to determine fatty acid profile. Fatty acids were transesterified following the method described by Shehata, de Man and Alexander (1970) with some modifications according to the procedure described by Domínguez, Borrajo and Lorenzo (2015a); 4 mL of a sodium methoxide (2) solution were added to the fraction, vortexed every 5 min during 20 min at room temperature, then 4 mL of a H2SO4 solution (in methanol at 50%), vortexed a few seconds and vortexed again before adding 2 mL of distilled water. Organic phase (containing fatty acids methyl esters) was extracted with 2.5 mL of hexane. Separation and quantification of the FAMEs was carried out using a gas chromatograph (GC-Agilent 6890N; Agilent Technologies Spain, S.L., Madrid, Spain) equipped with a flame ionization detector and an automatic sample injector HP 7683, and using a Supelco SPTM-2560 fused silica capillary column (100 m, 0.25 mm i. d., 0.2 μm film thickness). The chromatographic conditions were as follows: initial column temperature 120 °C, maintaining this temperature for 5 min, programmed to increase at a rate of 5 °C·min−1 up to 200 °C, maintaining this temperature for 2 min, then at 1 °C·min−1 up to 230 °C, maintaining this temperature for 3 min. The injector and detector were maintained at 260 and 280 °C, respectively. Helium was used as the carrier gas at a constant flow-rate of 1.1 mL·min−1, with the column head pressure set at 35.56 psi. The split ratio was 1:50 and 1 μL of solution was injected. Nonadecanoic acid (C19:0) at 0.3 mg·mL−1 was used as internal standard and added to the samples prior methylation. Individual FAMEs were identified by comparing their retention times with those of authenticated standards (Supelco 37 component FAME Mix). Data regarding FAME composition were expressed in percentage according to the weight of the total identified FAMEs. Data were expressed in g/100 g of fatty acid.

### 2.4 Statistical analysis

Data were analysed using the statistical package JMP^®^ Pro 13.1.0 by Copyright © 2016 SAS Institute Inc. The effect of species (sheep and goat), fat source (10% and 30% pork belly, or 10% and 30% olive oil), and their interaction were tested with a mixed model procedure, considering these variables as fixed effects (PROC MIXED, SAS). The residual random error associated with the observation has been considered. The predicted means obtained were ranked based on pair-wise least significance differences and compared using the t student test for *P < 0.05, **P < 0.01 or ***P < 0.001 significance levels.

## 3 Results and discussion

### 3.1 Physical characteristics

The effect of fat source on physical characteristics (pH, aw, dry matter and ashes) of sheep or goat pâtés is shown in Table 2. Significant differences were found between species, fat source and interactions on pH values. Pâtés incorporating olive oil had significantly lower pH comparing with pâtés with pork belly and the pâté made with sheep meat with 30% olive oil presented the lowest pH value. Even though there is an interaction between the species and the fat content for aw, the values range is small and vary between 0.96 and 0.98. Dry matter was significantly affected by fat source and pâtés made with sheep meat incorporating olive oil had higher dry matter percentage. An interaction between the fat source and species was detected and both goat and sheep pâtés incorporating olive oil presented a higher percentage of dry matter.

**Table 2:** Predicted values (means ± standard error) for fat source effect on physicochemical characteristics of sheep or goat pâtés.

	GPB1	GPB3	GOO1	GOO3	SPB1	SPB3	SOO1	SOO3	Significance		
									Species	Fat	Sp X F
**pH**	6.4 ± 0.02^a^	6.3 ± 0.02^a^	5.9 ± 0.02^c^	5.9 ± 0.02^c^	6.1 ± 0.02^b^	6.4 ± 0.02^a^	6.4 ± 0.02^a^	5.2 ± 0.08^d^	***	***	***
**a**_**W**_	0.96 ± 0.00^c^	0.96 ± 0.00^bc^	0.98 ± 0.00^a^	0.97 ± 0.00^ab^	0.97 ± 0.00^ab^	0.97 ± 0.00^bc^	0.96 ± 0.00^c^	0.96 ± 0.00^c^	ns	ns	*
**Dry Matter (%)**	41.4 ± 0.82^c^	39.3 ± 0.82^cd^	38.2 ± 0.82^de^	44.6 ± 0.82^b^	36.3 ± 0.82^e^	36.6 ± 0.82^e^	46.1 ± 0.82^ab^	48.3 ± 0.83^q^	ns	***	***
**Ashes (%)**	2.2 ± 0.17	2.2 ± 0.17	2.9 ± 0.17	2.2 ± 0.17	2.2 ± 0.17	2.0 ± 0.17	2.3 ± 0.17	2.4 ± 0.17	ns	ns	ns
**Protein (%)**	18.9 ± 0.17^d^	18.7 ± 0.17^d^	19.2 ± 0.17^cd^	19.6 ± 0.17^c^	22.3 ± 0.17^a^	21.9 ± 0.17^a^	20.7 ± 0.17^b^	21.0 ± 0.17^b^	***	***	***
**Collagen (%)**	1.34 ± 0.3	1.4 ± 0.3	1.54 ± 0.18	1.54 ± 0.18	1.30 ± 0.30	1.19 ± 0.30	1.54 ± 0.18	1.46 ± 0.17	ns	ns	ns
**Fat (%)**	9.7 ± 1.2^d^	13.6 ± 1.2^bcd^	11.4 ± 1.2^cd^	16.6 ± 1.2^ab^	14.0 ± 1.2^bc^	18.2 ± 1.2^a^	13.4 ± 1.2^bcd^	18.0 ± 1.2^a^	***	***	ns
**Cholesterol** (mg/100g sample)	26.0 ± 0.8^cd^	35.2 ± 2.6^a^	18.9 ± 0.8^f^	30.2 ± 0.8^b^	28.3 ± 0.8^bc^	37.4 ± 0.8^a^	22.9 ± 0.8^e^	23.2 ± 0.8^de^	ns	***	ns

Significance: ns: not significant; *P ≤ 0.05; **P ≤ 0.01; ***P ≤ 0.001; a ≠ b ≠ c ≠ d ≠ e ≠ f ≠ q for P ≤ 0.01.

**GPB1 (Goat + 10% pork belly); GPB3 (Goat + 30% pork belly); GOO1 (Goat + 10% olive oil); GOO2 (Goat + 30% olive oil).**

**SPB1 (Sheep + 10% pork belly); SPB3 (Sheep + 30% pork belly); SOO1 (Sheep + 10% olive oil); SOO2 (Sheep + 30% olive oil)**.

**Species (sheep, goat); Fat (pork belly, olive oil); Sp x F (interaction species x fat source)**.

The values found for pH and aw were close to those found for similar meat products as sheep or goat sausages (Leite et al., 2015), foal sausages (Lorenzo and Franco, 2012), sheep and goat “mantas” (Oliveira et al., 2014), sheep liver pâté (Amaral et al., 2013) or goat meat pâté (Dalmás et al., 2011).

### 3.2 Chemical composition

Table 2 also shows the effects of fat source and species on chemical composition and cholesterol content. Sheep pâtés had significantly higher protein and fat contents than goats. The pâtés incorporating 30% olive oil had the highest values of percentage of fat independently from the species, 16.6 and 18.0% for goats and sheep, respectively. However, although not significantly different from these pâtés was the sheep meat pâté with 30% of pork belly which presented the highest value of fat percentage (18.2%). No significant differences for collagen percentage were found among pâtés. The protein content found resembles the normal protein content of raw sheep or goat meats (Teixeira et al., 2015) ranging between 22 to 24%. However, the protein content found was higher than the protein content reported by Amaral et al. (2013) in sheep liver pâté (15.1%) and by Dalmás et al. (2011) in goat pâté (14.74%).

The low-fat content (9.7–18.2%) of the produced pâtés in comparison with other similar meat products is highlighted, particularly in sheep liver pâté (23.9%, Amaral et al., 2013) or goat pâté (22.67%, Dalmás et al., 2011). In our study the source of fat had a significant effect (P ≤ 0.001) on cholesterol content. Cholesterol content was lower in pâtés with olive oil (18.9 and 22.9 mg/100 g for sheep and goat pâtés with 10% olive oil, respectively). The highest values were found for sheep and goat with 30% pork belly (37.4 and 35.2 mg/100 g, respectively) and were similar to the values found by Domínguez et al. (2016) for pork pâté using olive oil as a back-fat replacer. In any case, the values found were lower than those indicated in the bibliography for other meats and meat products, 62 mg/100 g for pork meat (Parunovic et al., 2015) and values between 59 to 62 mg/200 g in chicken pâté (Polak et al., 2011).

### 3.3 Fatty acid profile

The fatty acid profile of the sheep or goat pâtés with more than 0.1g/100 g of fatty acids is shown in Table 3. The source (pork belly or olive oil) and proportion (10 or 30%) of fat affected significantly (p ≤ 0,05) the fatty acid profile.

**Table 3:** Predicted values (means ±standard error) for the fat source effect on fatty acid profile (expressed in g/100 g of fatty acids) of sheep or goat pâtés.

	GPB1	GPB3	GOO1	GOO3	SPB1	SPB3	SOO1	SOO3	Significance		
									Species	Fat	Sp. X F
**C10:0**	0.09 ± 0.02	0.03 ± 0.02	0.04 ± 0.02	0.1 ± 0.02	0.09 ± 0.02	0.01 ± 0.02	0.07 ± 0.02	0.03 ± 0.02	ns	ns	ns
**C12:0**	0.13 ± 0.03	0.05 ± 0.03	0.05 ± 0.03	0.13 ± 0.03	0.12 ± 0.03	0.16 ± 0.02	0.09 ± 0.03	0.05 ± 0.02	ns	ns	ns
**C14:0**	1.06 ± 0.27^abc^	0.51 ± 0.27^b^	0.68 ± 0.27^bc^	1.44 ± 0.27^ab^	1.41 ± 0.27^ab^	1.93 ± 0.27^c^	0.97 ± 0.27^bc^	0.50 ± 0.27^c^	ns	ns	*
**C15:0**	0.21 ± 0.02^bc^	0.12 ± 0.02^d^	0.15 ± 0.02^cd^	0.27 ± 0.02^ab^	0.27 ± 0.02^ab^	0.31 ± 0.02^ac^	0.22 ± 0.02^bc^	0.12 ± 0.02^d^	ns	ns	***
**C16:0**	16.5 ± 1.9^b^	14.8 ± 1.9^b^	16.2 ± 1.9^b^	20.1 ± 1.9^ab^	20.2 ± 1.9^ab^	24.8 ± 1.9^a^	16.4 ± 1.9^b^	14.8 ± 1.9^b^	ns	ns	*
**C16:1n-7**	0.92 ± 0.22^b^	0.76 ± 0.22^b^	0.84 ± 0.22^b^	1.36 ± 0.02^ab^	1.34 ± 0.22^ab^	1.74 ± 0.22^a^	0.91 ± 0.22^b^	0.73 ± 0.22^b^	ns	ns	*
**C17:0**	0.64 ± 0.06^bc^	0.32 ± 0.06^d^	0.50 ± 0.06^cd^	0.73 ± 0.06^ab^	0.73 ± 0.06^ab^	0.87 ± 0.06^a^	0.63 ± 0.06^bc^	0.34 ± 0.06^d^	ns	ns	***
**C17:1**	0.36 ± 0.02^a^	0.25 ± 0.02^c^	0.29 ± 0.02^bc^	0.35 ± 0.02^ab^	0.35 ± 0.02^ab^	0.34 ± 0.02^ab^	0.34 ± 0.02	0.24 ± 0.02^e^	ns	*	***
**C18:0**	8.8 ± 1.6^b c^	6.9 ± 1.6^c^	8.7 ± 1.6^bc^	13.1 ± 1.6^ab^	13.1 ± 1.6^ab^	17.3 ± 1.6^a^	9.4 ± 1.6^bc^	7.1 ± 1.6^c^	ns	ns	**
**C18:1n-9**	60.7 ± 4.5^ab^	65.9 ± 4.5^a^	50.8 ± 4.5^bc^	62.0 ± 4.5^ab^	50.8 ± 4.5b^c^	40.4 ± 4.5^c^	60.3 ± 4.5^ab^	66.0 ± 4.5^a^	ns	ns	**
**9t-C18:1**	1.03 ± 0.12^b^	0.36 ± 0.12^c^	0.91 ± 0.12^b^	1.29 ± 0.12^ab^	1.30 ± 0.12^ab^	1.46 ± 0.12^a^	1.05 ± 0.12^b^	0.36 ± 0.12^c^	ns	ns	***
**C18:2n-6**	7.0 ± 0.16^bc^	7.6 ± 0.16^a^	6.8 ± 0.16^c^	7.1 ± 0.08^abc^	7.1 ± 0.16^abc^	7.3 ± 0.16^abc^	6.9 ± 0.16^c^	7.4 ± 0.16^ab^	ns	*	ns
**C20:0**	0.27 ± 0.03 ^abcd^	0.34 ± 0.03^ab^	0.31 ± 0.03^abc^	0.25 ± 0.03^bcd^	0.24 ± 0.03^cd^	0.19 ± 0.03^d^	0.29 ± 0.03 ^abc^	0.35 ± 0.03^a^	ns	ns	**
**C20:1n-9**	0.18 ± 0.06^b^	0.20 ± 0.06^b^	0.19 ± 0.06^b^	0.35 ± 0.06^ab^	0.35 ± 0.02^ab^	0.49 ± 0.02^a^	0.20 ± 0.02^b^	0.20 ± 0.02^b^	ns	ns	*
**C18:3n-3**	0.86 ± 0.01^c^	0.76 ± 0.01^d^	0.92 ± 0.01^b^	0.96 ± 0.01^a^	0.94 ± 0.01^ab^	0.84 ± 0.01^c^	0.91 ± 0.01^b^	0.74 ± 0.01^d^	ns	***	***
**C21:0**	0.27 ± 0.02^a^	0.08 ± 0.02^b^	0.23 ± 0.02^a^	0.27 ± 0.02^a^	0.27 ± 0.02^a^	0.28 ± 0.02^a^	0.25 ± 0.02^a^	0.08 ± 0.02^b^	ns	**	***
**C20:3n-3**	0.47 ± 0.08^ab^	0.46±0-08^ab^	0.55 ± 0.08^a^	0.21 ± 0.08^bc^	0.22 ± 0.08^bc^	0.08±0-08^c^	0.43 ± 0.08^ab^	0.45 ± 0.08^ab^	ns	ns	*
**C20:4n-6**	0.23 ± 0.07	0.24 ± 0.07	0.22 ± 0.07	0.36 ± 0.07	0.36 ± 0.07	0.49 ± 0.07	0.22±0-07	0.24 ± 0.07	ns	ns	ns
**C20:5n-3**	0.08 ± 0.04^c^	0.08 ± 0.04^c^	0.16 ± 0.04^abc^	0.23 ± 0.04^ab^	0.23 ± 0.04^ab^	0.26 ± 0.04^a^	0.12 ± 0.04^bc^	0.08 ± 0.04^c^	ns	ns	**
**SFA**	28.1±a 3.8^bc^	23.3 ± 3.8^c^	27.0 ± 3.8^bc^	36.78 ± 3.8^ab^	36.5 ± 3.8^ab^	46.1 ± 3.8^a^	28.4 ± 3.8^bc^	23.5 ± 3.8^c^	ns	ns	**
**MUFA**	63.2 ± .4.1^ab^	67.5 ± 4.1^a^	64.3 ± 4.1^ab^	54.2 ± 4.1^bc^	54.3 ± 4.1^bc^	44.5 ± 4.1^c^	62.9 ± 4.1^ab^	67.6 ± 4.1^a^	ns	ns	**
**PUFA**	8.8 ± 0.28	9.2 ± 0.28	8.8 ± 0.28	9.2 ± 0.28	9.2 ± 0.28	9.5 ± 0.28	8.7 ± 0.28	9.0 ± 0.28	ns	ns	ns
**UFA**	71.9 ± 3.8^ab^	76.7 ± 3.8^a^	73.0 ± 3.8^ab^	63.4 ± 3.8^bc^	63.5 ± 3.8^bc^	53.9 ± 3.8^c^	71.6 ± 3.8^ab^	76.5 ± 3.8^a^	ns	ns	**
**P/S**	0.31 ± 0.02^c^	0.39 ± 0.02^a^	0.33 ± 0.02^bc^	0.26 ± 0.02^cd^	0.26 ± 0.02^cd^	0.21 ± 0.02^d^	0.30 ± 0.02^c^	0.38 ± 0.02^ab^	*	*	***
**PUFA n-3**	1.4 ± 0.03^b^	1.3 ± 0.02^c^	1.7 ± 0.03^a^	1.5 ± 0.03^b^	1.5 ± 0.03^b^	1.3 ± 0.03^c^	1.5 ± 0.03^b^	1.3 ± 0.03^c^	***	***	*
**PUFA n-6**	7.3 ± 0.3	7.8 ± 0.3	7.1 ± 0.3	7.8 ± 0.3	7.7 ± 0.3	8.2 ± 0.3	7.2±0-3	7.7 ± 0.3	ns	ns	ns
**PUFA n-6/n-3**	10.5 ± 0.2^cd^	10.3 ± 0.2^cd^	11.8 ± 0.2^a^	11.4 ± 0.2^ab^	11.3 ± 0.2^ab^	10.4 ± 0.2^cd^	10.8 ± 0.2^cd^	10.0 ± 0.2^d^	*	*	**

Significance: ns: not significant; *P ≤ 0.05; **P ≤ 0.01; ***P ≤ 0.001; a ≠ b ≠ c ≠ d for P ≤ 0.001. SFA: saturated fatty acids; MUFA: monounsaturated fatty acids; PUFA: polyunsaturated fatty acids; UFA: unsaturated fatty acids; P/S: PUFA/SFA; PUFA n-3: sum of PUFA of the n-3 series (C18:3n-3 + C20:3n-3 + C20:5n-3 + C22:6n-3); PUFA n-6: sum of PUFA of the n-6 series (C18:2n-6 + C18:3n-6 + C20:2n-6 + C20:3n-6 + C20:4n-6 + C22:2n-6); PUFA n-6/n-3: ratio of PUFAn-6/PUFAn-3.

**GPB1 (Goat + 10% pork belly); GPB3 (Goat + 30% pork belly); GOO1 (Goat + 10% olive oil); GOO2 (Goat + 30% olive oil).**

**SPB1 (Sheep + 10% pork belly); SPB3 (Sheep + 30% pork belly); SOO1 (Sheep + 10% olive oil); SOO2 (Sheep + 30% olive oil)**.

**Species (sheep, goat); Fat (pork belly, olive oil); Sp x F (interaction species x fat source)**.

In decreasing order of amount the major fatty acids in the pâtés were oleic (C18:1n-9**:** from 40.4 to 66 g/100 g of fatty acids), palmitic (C16:0; from 14.8 to 24.8 g/100 g of fatty acids), stearic (C18:0, from 6.9 to 17.3 g/100 g of fatty acids), linoleic (C18:2n-6; from 6.8 to 7.6 g/100 g of fatty acids), myristic (C14:0; from 0.5 to 1.9 g/100 g of fatty acids), and palmitoleic (C16:1 n-7; from 0.7 to 1.7 g/100 g of fatty acids) acids. The higher C18:1n-9 content of the pâtés incorporating olive oil, as well as the higher C18:0 and C18:2n-6 contents in pâtés with pork belly were expected given the fatty acids profiles of olive oil compared to the pork belly.

These findings agree with the fatty acid profile of sheep liver pâté reported by Amaral et al. (2013) and Leite et al. (2015) for sheep and goat meat sausages with different pork fat levels. It was detected between 0.36 and 1.46 g/100 g of fatty acids of elaidic acid (9t-C18:1). The value found was slightly higher than that found by Leite et al. (2015) for sheep and goat sausages, but contrary to the authors' study we did not find any amount of one other trans-fat identified as the major ruminant trans-fat, the vaccenic acid. This trans fatty acid (TFA), as well as other trans fats, is edible but its consumption has shown to increase the risk of coronary heart disease by several boards' committees of food and nutrition organizations. The elaidic acid used to be the main TFA isomer in industrial hydrogenation Mensink (2005) and according to Weggemans et al. (2004) the total amount of TFA in meat is lower and accounts ranging 2%–5% of the fatty acid content and the highest amount found was 1.46 g/100 g of fatty acids in pâté SPB3 (Sheep +30% pork belly). The population nutrient intake goal for TFA recommended by joint WHO/FAO expert consultation is less than 1% of total energy intake (Brouwer, 2016). Anyway, the World Health Organization (WHO) in 2009 Scientific Update on Trans fatty acids suggested that the intake of ruminant TFAs is low enough in most populations and not constitute a significant risk factor and according to Dhaka et al. (2011) ruminant animal products such as meat are rich in essential nutrients which are difficult to obtain from other sources and to ban these foods from human diet have detrimental effects on population particularly for infants’ nutrition.

MUFA refers to the major monounsaturated fatty acid found, that is the oleic acid, and PUFA states to the major polyunsaturated fatty acid found, the linoleic acid. Pâtés with olive oil are the ones that have significantly (P ≤ 0.001) lower saturated fat content (29.0 and 30.4 g/100 g of fatty acids for sheep and goat pâtés, respectively) and the goat pâtés with pork fat have generally a tendency although not significant to have less saturated fat proportion than sheep meat pâtés. As expected the pâtés with olive oil showed significantly (P ≤ 0.001) higher MUFA, 61.3 and 62.8 g/100 g of fatty acids, for goat and sheep meat pâtés, respectively. No significant differences were found for PUFA content which was varying between 8.7 and 9.5 g/100 g of fatty acids. The MUFA content found in the present study was higher than the MUFA content in the sheep liver pâté obtained by Amaral et al. (2013) and for sheep sausages with different pork lard contents by Bovolenta et al. (2008) or goat and sheep sausages by Leite et al. (2015). The PUFA content found is similar than the products of the studies cited before. The pâtés produced with a total fat percentage ranging 9.7–18.2 % majority UFA are according to the Food and Agriculture Organization of the United Nations (FAO, 2010) that are balanced foods increasing high density lipoprotein (HDL) cholesterol concentrations and reducing low density lipoprotein (LDL) concentrations with an interesting amount of linoleic acid indispensable sine that cannot be synthesized by humans.

The two major classes of PUFA are the omega-3 and omega-6 fatty acids. Several different omega-3 exist, but most of scientific research and health recommendations focus on three: the C18:3n-3 (alpha-linolenic acid known as ALA), the C20:5n-3 (eicosapentaenoic acid known as EPA), and the C22:6n-3 (docosahexaenoic acid known as DHA). Two of them, the ALA and EPA were detected although in proportions less than 0.1%. As expected the pâtés incorporating olive oil had a higher percentage of ALA. The most important Omega-6 fatty acid detected was the Linoleic acid (C18:2n-6) and in less amount the arachidonic acid (C20:4n-6). The presence of these fatty acids in the pâtés are important once according to the intake recommendations for fatty acids and other nutrients are provided in the Dietary Reference Intakes (DRIs) and the Estimated Average Requirement (EAR) developed by the Food and Nutrition Board of the Institute of Medicine (IOM) (Institute of Medicine, Food and Nutrition Board, 2005) which should be between 0.5 and 1.6 g/day depending the sex and age.

PUFA n-3 and PUFA n-6 contents varying between 1.3 to 1.7 and 7.1–8.2 g/100 g of fatty acids respectively are relatively higher than the values found by Leite et al. (2015) in sheep and goat meat sausages with different pork fat levels.

The ratios PUFA/SFA (P/S) and n-6/n-3, shown in Table 3 are indices widely used for several food and nutritional organizations to evaluate the nutritional value of fat in human diet and to make food recommendations.

The values of P/S ratio of the different pâtés in present study varied from 0.2 to 0.39. FAO (2010) recommended a P/S ratio between 0.4 and 0.5. The n-6/n-3 ratios observed in this study varying from 10 and 11.8 are relatively lower than the values (11.7–13.5) observed by Domínguez et al. (2016) for pork pâté using olive oil as a back-fat replacer but they are even higher than the ratio recommended which not exceed 4 according to Simopoulos (2004). However, according to WHO (2009) based on both the scientific evidence and conceptual limitations, there is no compelling scientific rationale for the recommendation of a specific ratio of n-6 to n-3 fatty acids or Linoleic acid (LA) to Alpha-linolenic acid (ALA).

## 4 Conclusions

The sheep and goat meat pâtés made with different fat proportions of pork belly fat or olive oil show to be an interest way to give value added to animals with very low commercial value. No significant differences for protein, fat or collagen percentages were found among pâtés. There was observed a low-fat content of the pâtés produced (9.7–18.2%) and a very high protein content ranging the 22–24% in comparison with other similar meat products. The source of fat (pork belly or olive oil) and the proportion of fat influenced significantly the fatty acid profile. Pâtés with olive oil are the ones that have lower saturated fat content and highest MUFA and goat meat pâtés have higher PUFA content.

The ratios P/S and n-6/n-3 of all pâtés show that are fat balanced products according to the most important food recommendations by world food and health organizations.

## Declaration

### Author contribution statement

Alfredo Teixeira, Samanta Almeida, Etelvina Pereira, Fernando Mangachaia, Sandra Rodrigues: Conceived and designed the experiments; Performed the experiments; Analyzed and interpreted the data; Contributed reagents, materials, analysis tools or data; Wrote the paper.

### Funding statement

This research did not receive any specific grant from funding agencies in the public, commercial, or not-for-profit sectors.

### Competing interest statement

The authors declare no conflict of interest.

### Additional information

No additional information is available for this paper.
